# The Book World of Medicine and Science

**Published:** 1905-04-15

**Authors:** 


### The Book World of Medicine and Science.

The Conjunctiva in Health and Disease. By N. Bishop Harman, M.A., M.B.Cantab., F.R.C.S., Ophthalmic Surgeon to the Belgrave Hospital for Children, etc. With numerous illustrations. (London: Baillière, Tindall, and Cox. 1905. 8vo., pp. 276. 10s. 6d.)

Mr. Harman describes his book on its title page as "a record of some research work," and to this description it is thoroughly well entitled. Its successive chapters deal, in most cases very completely, with the history of conjunctival affections as gathered from old writings, with the general anatomy of the membrane, with the ordinary age incidence of its diseases, and with the influence upon them of social conditions, with the statistics of blindness, as derived from the London County Council Schools, with the bacteriology of the conjunctiva and of its diseases, and with the varieties of the latter in considerable detail. As regards questions of treatment, the author has little that is new or encouraging to say. The cure of conjunctivitis, whether acute or chronic, does not appear to have been greatly promoted by the study of the micro-organisms by which it is caused or which are associated with it; partly perhaps because, generally speaking, applications which would be destructive to these organisms are too potent for the delicate membrane concerned. We have been surprised, in this relation, to find no mention of aqua chlori, which was much used by Von Graefe as a collyrium many years ago, and which was probably indebted to its bactericidal powers for any benefit which resulted from its employment. Mr. Harman differs from many modern writers in being very decided in his preference of the old-fashioned silver nitrate, in suitable solutions, to protargol or the other "colloid" preparations of silver, and he thinks the beneficial action which has been ascribed to protargol has been really due, in some instances, to the glycerine contained in the solution, and to the lacrymation produced by it, by which noxious organisms may have been washed away. We can hardly describe the book as being calculated to meet the demands of the meritorious person commonly described by authors as "the busy general practitioner," but it is an admirable account of all that can now be said to be known with regard to the important structure with which it deals, and it deserves cordial recognition as a piece of good and thorough work. We shall hope to meet with the author again, and with reference to some group of maladies more amenable to treatment.

Ambidexterity: or Two-Handedness and Two-Brainedness. By John Jackson, F.E.I.S. (London: Kegan Paul, Trench, Trübner and Co. 1905. Pp. 258.)

There is little doubt that systematic teaching of ambidexterity, or, we should say, rather, the prevention of single-handed development, in childhood would result in considerable benefit to the individuals concerned as well as to the community of which they are members. In the work before us the arguments in support of this proposition, as well as those which are adverse to it, are dealt with at length, and anyone who wishes to inquire into the matter will be well advised to obtain Mr. Jackson's book and read it for himself. Unfortunately the text is somewhat marred by the manner in which the author presents his arguments, and the reader is constantly irritated by alterations in the character of the type. The adult reader does not usually judge the value of a statement by the size of the type in which it is set out, and in a future edition Mr. Jackson would do well to omit this form of emphasis. There is one suggestion in the book against which we strongly protest, and that is as to the desirability of eaching children to perform simultaneous ambidextral work involving separate mental processes. Even if the hypothesis be tenable that ambidexterity renders it possible to carry on two different processes of thought at the same moment we cannot see that anything but harm can come from subjecting a child's brain to the endeavour. The eyes, in any case, can only look at one thing at a time, and the simultaneous writing of two different letters must of necessity involve a succession of rapid glances from one hand to the other; and it is probable that there is a similar alternation of thought from one object to another, with a consequent severe mental strain. In one of the examples which Mr. Jackson quotes this point is exemplified. A girl of sixteen who writes a letter to her mother with her left hand while writing to her father with her right hand says: "I am in agony just now trying to write two letters at the same time. It is not so easy as it looks." We quite believe her, and think it most undesirable that the attempt should be encouraged.

Health at School, Considered in its Mental, Moral, and Physical Aspects. By Clement Dukes, M.D., B.S.Lond., F.R.C.P.Lond., Physician to Rugby School. Fourth edition, revised, enlarged, and illustrated. (London: Rivingtons. 1905. 8vo. Pp. 606. Price 10s. 6d.)

A new edition of Dr. Clement Dukes's well known work scarcely calls for more than a few words of welcome from a reviewer, even although it contains the application, in constantly increasing minuteness of detail, of the sound principles which have always been advocated by its distinguished author. Dr. Dukes had the great merit of being one of the first to perceive that the education of the young should be regarded as a department of applied physiology, in which only those who had some knowledge of the principles of that science could properly claim to rank as experts; and his writings have always been based upon the sure foundation that bodily health must always underlie intellectual efficiency. He now teaches parents and schoolmasters how this essential bodily health may be attained and preserved under the conditions which commonly exist in educational establishments, what evils incidental to these conditions are to be expected, and how, in the majority of instances, they may be obviated or removed. It would be impossible within our limits to do any justice either to the principles which Dr. Dukes lays down or to the practical applications which he derives from them; and it would be invidious, where all is excellent, to select passages for particular commendation. We can only counsel parents to read the book carefully for themselves; and, in the selection of schools for their children, to be largely guided by the advice which it contains.

Infantile Mortality and Infant Milk Depôts. By G. F. McCleary, M.D., D.P.H., Medical Officer of Health of the Metropolitan Borough of Battersea. (London: P. S. King & Son, 1905. Pp. 135. Price 6s. net.)

Dr. McCleary is well known as one of the pioneers in this country of milk reform, and he has done well to place before the public in book form the numerous factors which bear upon a many-sided national problem. Commencing with a chapter on the decline in the English birth-rate, which emphasises the importance of the questions which follow, the author continues with some chapters on the causes of infantile mortality, and quotes statistics which show that a very large part of this mortality is due to improper food. Like every one else who has studied the matter, Dr. McCleary insists that breast feeding is the only proper way of supplying nutriment to the young baby. There is no substitute for the mother's milk. Nevertheless, for economic and physiological reasons, breast feeding is impossible in a great proportion of cases, and some sort of substitute has to be found. Pro-

### THE BOOK WORLD OF MEDICINE AND SCIENCE—*Continued*

prietary foods the author condemns on the ground that, generally speaking, they are deficient in fat, too rich in carbohydrates, and lacking in the anti scorbutic elements. Cow's milk he regards as the chief and best substitute. But, with cow's milk there is the objection that by the time it comes to the consumer it is grossly contaminated with filth and micro-organisms, which, especially in warm weather, may set up fatal illness in the infant who swallows the milk. There are two measures which may be taken to avoid this, namely, prevention of contamination during collection and distribution of the milk, and sterilisation of the milk at central depôts before passing it on to the consumer. Dr. McCleary dismisses the former plan as beyond the range of practical politics. He urges that it would be too costly, and that it would fail to solve the problem, because much of the contamination occurs after the milk has reached the consumer. Therefore, he is prepared to rely upon the milk depôt system, combined with suitable instruction in hygiene to girls and young mothers of the lower classes, as our future safeguards against excessive infantile mortality from diarrhœal complaints. The high rate of mortality among infants at the present day is a blot upon our national economy, which it must be the earnest wish of every one, who fully realises the duties of citizenship, to remedy, and in this book Dr. McCleary has provided a ready means by which medical and lay men alike can furnish themselves with the knowledge requisite for the establishment of reform. We strongly recommend the work.

The Preparation and After-Treatment of Section Cases By W. J. Stewart McKay, M.B., M.Ch., Senior Surgeon to the Lewisham Hospital for Women and Children, Sydney. (London: Baillière, Tindall and Cox. 1905. Pp. 651. Price 15s.)

Mr. McKay has not, we think, been quite happy in the choice of a title for his book, which deals only with cases of abdominal section, and is based chiefly upon gynæcological experience. But there is very little fault to find with the contents. The author is a master of detail, and, moreover, has succeeded in presenting minutiæ in such a way that they are not wearisome to read. In the first portion of the book there is a suggestion to the effect that the surgeon should keep a "consent book," in which every patient about to be operated upon must enter a signed permission for the operation. Although instances of recrimination such as the "consent book" is intended to avoid are not common, yet they have occurred, and have resulted in troublesome litigation, and Mr. McKay's advice is therefore worthy of notice. He insists very rightly on the desirability of keeping a patient on whom it is proposed to perform an abdominal section under observation for several days previous to operation. If this rule be not observed, occasionally a life will be lost from some unsuspected associated disease. In the chapter on post-operation vomiting there is a statement to which we must take exception. Mr. McKay states that "little or nothing can be done, or need be done, for the vomiting that takes place during the first three or four hours. We cannot stop it, and we cannot prevent it." As a matter of fact washing out the stomach with warm water before the patient is removed from the operating table will usually prevent post-operation nausea and vomiting. In the chapter on tympanites Mr. McKay has made extensive inroads into the realms of hypothesis without any material profit, and we suggest that in a future edition this particular chapter may be curtailed with advantage. On the whole, we are able to congratulate Mr. McKay upon the production of a work which will prove of great value to everyone upon whom may fall the responsibility of the preparation and after-treatment of patients who have to undergo abdominal section.

International Clinics. Vol. IV. Fourteenth Series. Edited by A. O. J. Kelly, M.D. (Philadelphia and London: J. B. Lippincott Company. 1905. Pp. 314. With Illustrations. Price 10s. 6d.)

This volume of international clinics, like its predecessors, contains many valuable contributions by eminent men, practising in various countries. The book is arranged in sections under the headings Treatment, Medicine, Surgery, Gynæcology, Neurology, and Pathology. The following is a list of contributors—George Hayem, Adolphe Javal, Myrom Metzenbaum, F. Lejars, F. Parkes Weber and J. H. Watson, S. Solis Cohen, Dyce Duckworth, H. Senator, Alexander Crombie, Robert Dawson Rudolf, E. H. Bradford, Wisner R. Townsend, J. Lincoln Porter, J. K. Young, W. Arbuthnot Lane, Anthony A. Bowlby, A. E. Gallant, F. A. L. Lockhart, D. R. Brower, A. S. Warthin, and C. F. Craig. A glance through this list of names will be a sufficient guarantee of the high standard and value of the contents. Several of the papers contain the results of original observations and research, and therefore have a pressing claim for attention, while there is not one that is not worthy of careful perusal.

The Harmsworth Encyclopædia. Parts I., II., and III. (London: The Amalgamated Press, Limited, and Thomas Nelson and Sons. In 40 fortnightly parts. Price 7d. for each part.)

We have received the first three parts of this encyclopædia, and if they may be taken as a fair sample of the whole production we may safely anticipate for it a great success. The descriptions are lucid, up to date, and sufficiently copious for all purposes for which an encyclopædia is required. The medical articles are simple and appropriate, and it is evident that much care has been bestowed upon their composition.

The Naked Eye Anatomy of the Human Teeth. By T. Constant. (Bristol: J. Wright and Co.)

It would be difficult to find a more complete and exact account of the anatomy of the individual teeth and soft parts of the mouth than is contained in Mr. Constant's book, and it should at once appeal to the student as a most useful text-book for examination purposes. One is, perhaps, disappointed with a certain lack of originality, but no pains have been spared to quote other authors whose works deal with the subject in question.

### BOOKS RECEIVED.

T. Fisher Unwin.

"Studies in General Physiology." Two Vols. By Jacques Loeb.

Rebman, Limited.

"Tropical Light." By Major Chas. E. Woodruff, M.D.

Baillière, Tindall and Cox.

